# Diaqua­bis(ethyl­enediamine-κ^2^ *N*,*N*′)copper(II) 2,2′-dithio­dinicotinate sesquihydrate

**DOI:** 10.1107/S1600536809022612

**Published:** 2009-06-20

**Authors:** Turan Kaya Yazicilar, Serkan Demir, Ibrahim Uçar, Canan Kazak

**Affiliations:** a Ondokuz May˙is University, Art and Science Faculty, Department of Chemistry, 55139 Samsun, Turkey; b Ondokuz May˙is University, Art and Science Faculty, Department of Physics, 55139 Samsun, Turkey

## Abstract

In the title compound, [Cu(C_2_H_8_N_2_)_2_](C_12_H_6_N_2_O_4_S_2_)·1.5H_2_O, there are two half-molecules of the cationic complex in the asymmetric unit. The Cu^2+^ ions lie on inversion centres and are octa­hedrally coordinated by two ethyl­enediamine (en) and two aqua ligands in a typical Jahn–Teller distorted environment with the water O atoms in the axial positions. Two 2-mercaptonicotinate units (mnic) are linked by a disulfide bridge. All the ethyl­enediamine N—H and O—H groups form inter­molecular hydrogen bonds with acceptor O and N atoms, giving rise to a three-dimensional network. One of the uncoordinated water molecules has a site occupation factor of 0.5.

## Related literature

For the oxidation of thiols to disulfides, see: Yiannos & Karaninos (1963 ▶); Chowdhury *et al.* (1994 ▶); Yamamoto & Sekine (1984 ▶). For metal-organic disulfide salts, see: Briansó *et al.* (1981 ▶); Casals *et al.* (1987 ▶). For related structures, see: Kazak *et al.* (2004 ▶); Harrison *et al.* (2007 ▶). Cargill Thompson *et al.* (1997 ▶). 
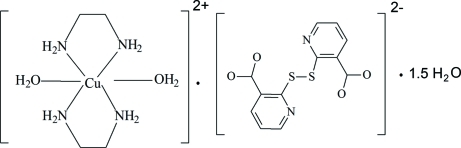


## Experimental

### 

#### Crystal data

[Cu(C_2_H_8_N_2_)_2_](C_12_H_6_N_2_O_4_S_2_)·1.5H_2_O
*M* *_r_* = 552.14
Triclinic, 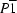

*a* = 8.8302 (9) Å
*b* = 11.5975 (11) Å
*c* = 11.7132 (11) Å
α = 95.800 (8)°
β = 101.703 (8)°
γ = 93.493 (8)°
*V* = 1164.5 (2) Å^3^
*Z* = 2
Mo *K*α radiation
μ = 1.17 mm^−1^
*T* = 297 K
0.35 × 0.20 × 0.15 mm

#### Data collection

Stoe IPDS-2 diffractometer
Absorption correction: integration (*X-RED*; Stoe & Cie, 2002 ▶) *T*_min_ = 0.540, *T*_max_ = 0.751
17957 measured reflections
4964 independent reflections
4034 reflections with *I* > 2σ(*I*)
*R*_int_ = 0.082

#### Refinement

*R*[*F* ^2^ > 2σ(*F* ^2^)] = 0.034
*wR*(*F* ^2^) = 0.088
*S* = 1.02
4964 reflections
333 parameters
6 restraints
H atoms treated by a mixture of independent and constrained refinement
Δρ_max_ = 0.51 e Å^−3^
Δρ_min_ = −0.69 e Å^−3^

### 

Data collection: *X-AREA* (Stoe & Cie, 2002 ▶); cell refinement: *X-AREA*; data reduction: *X-RED* (Stoe & Cie, 2002 ▶); program(s) used to solve structure: *SHELXS97* (Sheldrick, 2008 ▶); program(s) used to refine structure: *SHELXL97* (Sheldrick, 2008 ▶); molecular graphics: *ORTEPIII* (Burnett & Johnson, 1996 ▶); software used to prepare material for publication: *WinGX* (Farrugia, 1999 ▶) and *PLATON* (Spek, 2009 ▶).

## Supplementary Material

Crystal structure: contains datablocks I, global. DOI: 10.1107/S1600536809022612/hg2515sup1.cif

Structure factors: contains datablocks I. DOI: 10.1107/S1600536809022612/hg2515Isup2.hkl

Additional supplementary materials:  crystallographic information; 3D view; checkCIF report

## Figures and Tables

**Table 1:** Selected bond lengths (Å)

Cu1—N1	2.0053 (19)
Cu1—N2	2.0155 (18)
Cu2—N3	2.0148 (19)
Cu2—N4	2.0248 (18)
Cu1—O1*W*	2.702 (2)
Cu2—O2*W*	2.499 (2)

**Table 2:** Hydrogen-bond geometry (Å, °)

*D*—H⋯*A*	*D*—H	H⋯*A*	*D*⋯*A*	*D*—H⋯*A*
N1—H1*A*⋯O2^i^	0.90	2.25	3.084 (3)	154
N1—H1*B*⋯O3	0.90	2.48	3.138 (3)	130
N2—H2*A*⋯O3*W*^ii^	0.90	2.38	3.213 (3)	154
N2—H2*B*⋯O4^iii^	0.90	2.59	3.345 (4)	142
N4—H4*B*⋯O2^iv^	0.90	2.27	3.116 (3)	157
O1*W*—H2*W*⋯O2^v^	0.847 (17)	1.925 (18)	2.771 (2)	175 (3)
O1*W*—H1*W*⋯O3*W*	0.803 (17)	2.095 (18)	2.892 (3)	172 (3)
O2*W*—H3*W*⋯O4^iii^	0.820 (18)	1.95 (2)	2.712 (3)	154 (4)
O2*W*—H4*W*⋯O1^vi^	0.830 (17)	2.079 (18)	2.897 (3)	168 (3)
O3*W*—H5*W*⋯O1^vi^	0.828 (18)	2.025 (19)	2.838 (3)	167 (3)
O3*W*—H6*W*⋯O3^vii^	0.841 (18)	1.980 (19)	2.812 (2)	170 (3)
N3—H3*A*⋯O4*W*	0.87 (4)	2.42 (3)	3.045 (4)	129 (3)

Symmetry codes: (i) 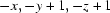; (ii) 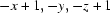; (iii) 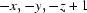; (iv) 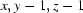; (v) 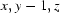; (vi) 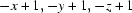; (vii) 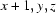.

**Fig. 1.**
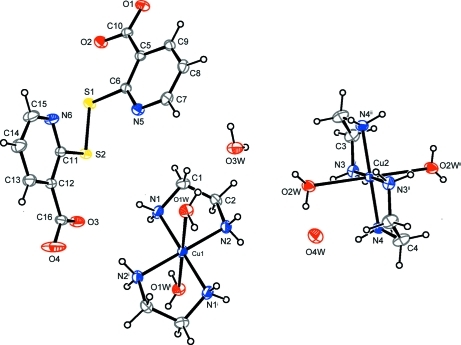
: ORTEPIII (Burnett & Johnson, 1996) plot of the copper(II) complex. Non-H atoms are drawn with displacement ellipsoids at the 30% probability level and H atoms are shown as small spheres of arbitrary radii. Water molecules are omitted for the clarity. [Symmetry codes: (i) -x, -y, 1 - z; (ii) 1 - x, -y, -z]

**Fig. 2.**
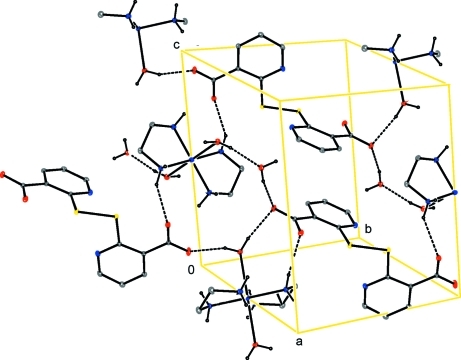
: Showing of intermolecular hydrogen bonding interactions (dashed lines) in the unitcell.

## supplementary crystallographic information

### Comment

As is well known, many oxidizing agents, such as nitric acid, hydrogen peroxide, oxygen, dimethyl sulfoxide and potassium ferricyanide, can oxidize thiols to disulfides (Yiannos & Karaninos, 1963). In several cases, the thiol-to-disulfide conversion can also be quickly completed *via* oxygen in the presence of certain metal ions (Chowdhury *et al.*, 1994; Yamamoto & Sekine, 1984). In the present case, the formation of the mnic-mnic (mnic: 2-mercaptonicotinate) dianion may be due to the oxidation of mnic *via* oxygen in the presence of Cu(II). It was of interest to determine the structure of the title compound, as there are a limited number of documented metal-organic disulfide salts (Briansó *et al.*, 1981; Casals *et al.*, 1987). Here, we report the crystal structure of the title compound, (I).

The asymmetric unit of compound (I) contains two crystallographically independent half-complexes in which the ethylenediamine (en) ligands, aqua ligands, 2-mercaptonicotinate anions and water molecules occupy general positions, whereas the Cu(II) ions are located on centres of inversion. In the crystal structure of the title compound, (I), the Cu(II) ions are coordinated by four N atoms of en ligands, forming a slightly distorted square plane. The Cu—N distances of 2.005 (2), 2.016 (2), 2.025 (2), and 2.015 (2)Å are comparable to those in other ethylenediamine-copper(II) complexes, such as *trans*-Bis(ethylenediamine)bis(*p*-nitrobenzoxasulfamato)copper(II) (Kazak *et al.*, 2004), Diaquabis(ethylenediamine) copper(II) bis(4-nitrobenzoate) (Harrison *et al.*, 2007), The coordination sphere of the Cu(II)ions is completed by two longer contacts to two symmetry equivalent aqua ligands located above and below the tetragonal plane. The Cu—Ow distances of 2.702 (2)Å (Cu1—O1) and 2.499 (2)Å (Cu2—O2) are strongly elongated due to Jahn-Teller distortion and the coordination polyhedra around the Cu(II) ions can be described as significantly distorted octahedral.

The mnic-mnic dianion acts as a counter anion in title compound. The torsion angle about the S—S bond [C6—S1—S2—C11] is 81.98 (9)°, which is larger than those reported in L—*L* (76.5°) [Ag(L—*L*)](PF_6_) {L—*L*= 2,2'-bis[6-(2,2'-bipyridyl)]diphenyldisulfide, (Cargill Thompson *et al.*, 1997)}. The S—S bond length is 2.0352 (8) Å, which is comparable with those observed in [C_5_H_9_NH(CH_3_)S]_2_[CuCl_4_] [2.02 (2) Å; (Briansó  *et al.*, 1981)], [{(CH_3_)_2_NH(CH_2_)_3_S}_2_] [CdBr_4_] [2.013 (3) Å; (Casals *et al.*, 1987)].

The crystal packing of (I) is formed *via* interesting intermolecular hydrogen bonding interactions. It can be seen from Fig. 2 that two complex cations and two dianions are joined to each other by N—H···O and O—H···O hydrogen bonds (Table 2), which lead to three dimensional extended network in the unitcell.

### Experimental

2-mercaptonicotinic acid (0.31 g, 2 mmol) (HMNA) was added into a solution of Cu(II)Cl_2_.2H_2_O (0.17 g, 1 mmol) in ethanol (40 ml). After stirring for 30 min, ethylenediamine (0.12 g, 2 mm l) was added into solutions of these compounds, under stirring, and mixtures were allowed to stand at room temperature. After a few days, well formed purple crystals were selected for X-ray studies.

### Refinement

H atoms attached to C and ethylenediamine N atoms were placed at calculated positions (C—H=0.93, 0.97 Å; N—H= 0.90 Å) and were allowed to ride on the parent atom [*U*_iso_(H)=1.2_eq_(C) and *U*_iso_(H)=1.2_eq_(N)]. The remaining H atoms were located in a difference map. At this stage, the maximum difference density of 3.76 e Å^-3^ indicated the presence of a possible atom site. A check of the solvent-accessible volume using *PLATON* (Spek, 2009) showed a total potential volume of 14.6 Å^3^. Attempts to refine this peak as a water O atom (O4W) resulted in a partial occupancy of 0.5. H atoms attached to O4W were not located.

### Crystal data

**Table:**

[Cu(C_2_H_8_N_2_)_2_](C_12_H_6_N_2_O_4_S_2_)·1.5H_2_O	*Z* = 2
*M**_r_* = 552.14	*F*(000) = 574.0
Triclinic, *P*1	*D*_x_ = 1.575 Mg m^−^^3^
Hall symbol: -P 1	Mo *K*α radiation, λ = 0.71069 Å
*a* = 8.8302 (9) Å	Cell parameters from 12659 reflections
*b* = 11.5975 (11) Å	θ = 1.8–27.0°
*c* = 11.7132 (11) Å	µ = 1.17 mm^−^^1^
α = 95.800 (8)°	*T* = 297 K
β = 101.703 (8)°	Prism, blue
γ = 93.493 (8)°	0.35 × 0.20 × 0.15 mm
*V* = 1164.5 (2) Å^3^	

### Data collection

**Table:**

Stoe IPDS-2 diffractometer	4964 independent reflections
Radiation source: fine-focus sealed tube	4034 reflections with *I* > 2σ(*I*)
graphite	*R*_int_ = 0.082
Detector resolution: 6.67 pixels mm^-1^	θ_max_ = 26.8°, θ_min_ = 1.8°
ω scans	*h* = −11→11
Absorption correction: integration (*X-RED*; Stoe & Cie, 2002)	*k* = −14→14
*T*_min_ = 0.540, *T*_max_ = 0.751	*l* = −14→14
17957 measured reflections	

### Refinement

**Table:**

Refinement on *F*^2^	Primary atom site location: structure-invariant direct methods
Least-squares matrix: full	Secondary atom site location: difference Fourier map
*R*[*F*^2^ > 2σ(*F*^2^)] = 0.034	Hydrogen site location: inferred from neighbouring sites
*wR*(*F*^2^) = 0.088	H atoms treated by a mixture of independent and constrained refinement
*S* = 1.02	*w* = 1/[σ^2^(*F*_o_^2^) + (0.052*P*)^2^] where *P* = (*F*_o_^2^ + 2*F*_c_^2^)/3
4964 reflections	(Δ/σ)_max_ < 0.001
333 parameters	Δρ_max_ = 0.51 e Å^−^^3^
6 restraints	Δρ_min_ = −0.69 e Å^−^^3^

### Special details

**Table:**

Geometry. All e.s.d.'s (except the e.s.d. in the dihedral angle between two l.s. planes) are estimated using the full covariance matrix. The cell e.s.d.'s are taken into account individually in the estimation of e.s.d.'s in distances, angles and torsion angles; correlations between e.s.d.'s in cell parameters are only used when they are defined by crystal symmetry. An approximate (isotropic) treatment of cell e.s.d.'s is used for estimating e.s.d.'s involving l.s. planes.
Refinement. Refinement of *F*^2^ against ALL reflections. The weighted *R*-factor *wR* and goodness of fit *S* are based on *F*^2^, conventional *R*-factors *R* are based on *F*, with *F* set to zero for negative *F*^2^. The threshold expression of *F*^2^ > σ(*F*^2^) is used only for calculating *R*-factors(gt) *etc*. and is not relevant to the choice of reflections for refinement. *R*-factors based on *F*^2^ are statistically about twice as large as those based on *F*, and *R*- factors based on ALL data will be even larger.

### Fractional atomic coordinates and isotropic or equivalent isotropic displacement parameters (Å^2^)

**Table:**

	*x*	*y*	*z*	*U*_iso_*/*U*_eq_	Occ. (<1)
Cu1	0.0000	0.0000	0.5000	0.04125 (11)	
Cu2	0.5000	0.0000	0.0000	0.03352 (10)	
C1	0.1188 (3)	0.2239 (2)	0.4661 (2)	0.0450 (5)	
H1C	0.2207	0.2211	0.5155	0.054*	
H1D	0.1026	0.3042	0.4548	0.054*	
C2	0.1061 (3)	0.1519 (2)	0.3502 (2)	0.0423 (5)	
H2C	0.0080	0.1608	0.2982	0.051*	
H2D	0.1896	0.1764	0.3132	0.051*	
C3	0.4528 (3)	0.2196 (2)	−0.0834 (3)	0.0594 (7)	
H3C	0.3833	0.2806	−0.0981	0.071*	
H3D	0.5142	0.2150	−0.1433	0.071*	
C4	0.4437 (4)	−0.2470 (2)	−0.0345 (3)	0.0633 (7)	
H4C	0.5049	−0.2639	−0.0933	0.076*	
H4D	0.3731	−0.3147	−0.0356	0.076*	
C5	0.4242 (2)	0.61714 (17)	0.65562 (17)	0.0306 (4)	
C6	0.3127 (2)	0.53935 (17)	0.68329 (17)	0.0311 (4)	
C7	0.4656 (3)	0.3882 (2)	0.6739 (2)	0.0415 (5)	
H7	0.4796	0.3101	0.6798	0.050*	
C8	0.5823 (2)	0.4564 (2)	0.6463 (2)	0.0418 (5)	
H8	0.6735	0.4257	0.6346	0.050*	
C9	0.5602 (2)	0.5710 (2)	0.63653 (18)	0.0366 (4)	
H9	0.6371	0.6189	0.6169	0.044*	
C10	0.4048 (2)	0.74330 (18)	0.64540 (18)	0.0343 (4)	
C11	0.0755 (2)	0.40673 (18)	0.86336 (18)	0.0331 (4)	
C12	0.0124 (2)	0.30855 (19)	0.90351 (18)	0.0358 (4)	
C13	0.0685 (3)	0.2922 (2)	1.0193 (2)	0.0470 (5)	
H13	0.0294	0.2284	1.0496	0.056*	
C14	0.1820 (3)	0.3696 (2)	1.0903 (2)	0.0544 (6)	
H14	0.2197	0.3598	1.1685	0.065*	
C15	0.2366 (3)	0.4609 (2)	1.0415 (2)	0.0538 (6)	
H15	0.3140	0.5130	1.0886	0.065*	
C16	−0.1116 (3)	0.2227 (2)	0.8278 (2)	0.0442 (5)	
N1	−0.0025 (2)	0.17358 (16)	0.52101 (17)	0.0433 (4)	
H1A	−0.0961	0.1943	0.4869	0.052*	
H1B	0.0163	0.2000	0.5979	0.052*	
N2	0.1161 (2)	0.02998 (17)	0.37308 (17)	0.0422 (4)	
H2A	0.2162	0.0158	0.3960	0.051*	
H2B	0.0746	−0.0176	0.3071	0.051*	
N3	0.3630 (2)	0.10872 (18)	−0.08804 (19)	0.0421 (4)	
N4	0.3548 (2)	−0.14487 (17)	−0.06077 (17)	0.0417 (4)	
H4A	0.2733	−0.1449	−0.0254	0.050*	
H4B	0.3189	−0.1473	−0.1387	0.050*	
N5	0.3322 (2)	0.42816 (16)	0.69298 (18)	0.0401 (4)	
N6	0.1866 (2)	0.48064 (17)	0.93068 (18)	0.0454 (4)	
O1	0.51047 (19)	0.80272 (15)	0.61677 (16)	0.0499 (4)	
O2	0.28373 (18)	0.78292 (14)	0.66900 (16)	0.0470 (4)	
O1W	0.2788 (2)	0.01895 (16)	0.65012 (16)	0.0467 (4)	
O3	−0.16845 (19)	0.24297 (16)	0.72768 (16)	0.0533 (4)	
O2W	0.3687 (2)	0.04649 (18)	0.16705 (16)	0.0535 (4)	
O4	−0.1483 (3)	0.1345 (2)	0.8711 (2)	0.1045 (11)	
O3W	0.5707 (2)	0.10145 (16)	0.59883 (17)	0.0509 (4)	
S1	0.13210 (6)	0.58824 (5)	0.70793 (5)	0.03837 (13)	
S2	0.00794 (5)	0.43656 (5)	0.71566 (5)	0.03637 (13)	
O4W	0.0516 (5)	−0.0045 (4)	−0.0659 (4)	0.0690 (11)	0.50
H1W	0.361 (2)	0.047 (2)	0.642 (2)	0.044 (7)*	
H2W	0.286 (3)	−0.0529 (16)	0.656 (3)	0.054 (8)*	
H3W	0.317 (4)	−0.012 (2)	0.176 (3)	0.088 (12)*	
H4W	0.403 (3)	0.081 (2)	0.2339 (17)	0.048 (7)*	
H5W	0.547 (3)	0.119 (3)	0.5309 (18)	0.062 (9)*	
H6W	0.641 (3)	0.149 (2)	0.640 (3)	0.069 (10)*	
H3B	0.323 (3)	0.081 (2)	−0.162 (3)	0.048 (7)*	
H3A	0.284 (4)	0.121 (3)	−0.056 (3)	0.081 (11)*	

### Atomic displacement parameters (Å^2^)

**Table:**

	*U*^11^	*U*^22^	*U*^33^	*U*^12^	*U*^13^	*U*^23^
Cu1	0.0621 (2)	0.02856 (19)	0.0401 (2)	0.00822 (16)	0.02562 (18)	0.00415 (15)
Cu2	0.03532 (18)	0.02982 (18)	0.03488 (19)	−0.00104 (13)	0.00566 (14)	0.00699 (14)
C1	0.0421 (11)	0.0343 (11)	0.0562 (14)	0.0004 (9)	0.0045 (10)	0.0070 (10)
C2	0.0387 (11)	0.0446 (13)	0.0486 (13)	0.0060 (9)	0.0162 (9)	0.0139 (10)
C3	0.0743 (17)	0.0412 (14)	0.0640 (17)	0.0082 (12)	0.0107 (14)	0.0180 (12)
C4	0.0822 (19)	0.0350 (13)	0.0671 (18)	−0.0033 (12)	0.0051 (15)	0.0062 (12)
C5	0.0314 (9)	0.0326 (10)	0.0256 (9)	−0.0009 (7)	0.0020 (7)	0.0026 (7)
C6	0.0297 (9)	0.0316 (10)	0.0314 (10)	0.0019 (7)	0.0052 (7)	0.0042 (8)
C7	0.0451 (11)	0.0346 (11)	0.0446 (12)	0.0138 (9)	0.0059 (9)	0.0044 (9)
C8	0.0324 (10)	0.0526 (13)	0.0391 (11)	0.0126 (9)	0.0043 (8)	0.0000 (10)
C9	0.0287 (9)	0.0478 (12)	0.0310 (10)	−0.0022 (8)	0.0045 (7)	0.0006 (9)
C10	0.0369 (10)	0.0329 (10)	0.0305 (10)	−0.0030 (8)	0.0027 (8)	0.0043 (8)
C11	0.0290 (9)	0.0314 (10)	0.0378 (11)	−0.0007 (7)	0.0081 (8)	−0.0014 (8)
C12	0.0360 (10)	0.0352 (11)	0.0348 (10)	−0.0062 (8)	0.0094 (8)	−0.0016 (8)
C13	0.0566 (13)	0.0442 (13)	0.0379 (12)	−0.0096 (10)	0.0095 (10)	0.0034 (10)
C14	0.0611 (14)	0.0569 (16)	0.0374 (12)	−0.0071 (12)	−0.0023 (11)	0.0015 (11)
C15	0.0542 (13)	0.0481 (14)	0.0476 (14)	−0.0138 (11)	−0.0055 (11)	−0.0054 (11)
C16	0.0495 (12)	0.0442 (13)	0.0366 (12)	−0.0186 (10)	0.0123 (9)	−0.0001 (9)
N1	0.0597 (11)	0.0339 (10)	0.0393 (10)	0.0088 (8)	0.0172 (8)	0.0013 (8)
N2	0.0476 (10)	0.0399 (10)	0.0429 (10)	0.0075 (8)	0.0186 (8)	0.0027 (8)
N3	0.0464 (10)	0.0428 (11)	0.0381 (11)	0.0088 (8)	0.0084 (9)	0.0072 (8)
N4	0.0453 (9)	0.0402 (10)	0.0384 (10)	−0.0057 (8)	0.0095 (8)	0.0035 (8)
N5	0.0393 (9)	0.0307 (9)	0.0519 (11)	0.0060 (7)	0.0109 (8)	0.0082 (8)
N6	0.0446 (10)	0.0380 (10)	0.0469 (11)	−0.0096 (8)	0.0008 (8)	−0.0016 (8)
O1	0.0482 (9)	0.0410 (9)	0.0621 (11)	−0.0096 (7)	0.0154 (8)	0.0137 (8)
O2	0.0450 (8)	0.0341 (8)	0.0657 (11)	0.0056 (7)	0.0161 (7)	0.0133 (8)
O1W	0.0489 (10)	0.0412 (10)	0.0501 (10)	0.0074 (8)	0.0079 (8)	0.0088 (8)
O3	0.0520 (9)	0.0542 (11)	0.0454 (10)	−0.0206 (8)	−0.0011 (7)	0.0047 (8)
O2W	0.0598 (10)	0.0607 (12)	0.0392 (9)	−0.0194 (9)	0.0190 (8)	−0.0004 (8)
O4	0.142 (2)	0.0901 (18)	0.0569 (13)	−0.0833 (17)	−0.0166 (13)	0.0258 (12)
O3W	0.0565 (10)	0.0467 (10)	0.0447 (10)	−0.0127 (8)	0.0032 (8)	0.0080 (8)
S1	0.0325 (2)	0.0305 (3)	0.0555 (3)	0.00462 (19)	0.0141 (2)	0.0103 (2)
S2	0.0304 (2)	0.0362 (3)	0.0411 (3)	−0.00393 (19)	0.00569 (19)	0.0055 (2)
O4W	0.060 (2)	0.073 (3)	0.076 (3)	0.007 (2)	0.016 (2)	0.017 (2)

### Geometric parameters (Å, °)

**Table:**

Cu1—N1	2.0053 (19)	C10—O1	1.246 (2)
Cu1—N2	2.0155 (18)	C10—O2	1.259 (3)
Cu2—N3	2.0148 (19)	C11—N6	1.329 (3)
Cu2—N4	2.0248 (18)	C11—C12	1.402 (3)
Cu1—O1W	2.702 (2)	C11—S2	1.788 (2)
Cu2—O2W	2.499 (2)	C12—C13	1.382 (3)
C1—N1	1.476 (3)	C12—C16	1.508 (3)
C1—C2	1.501 (4)	C13—C14	1.380 (3)
C1—H1C	0.9700	C13—H13	0.9300
C1—H1D	0.9700	C14—C15	1.362 (4)
C2—N2	1.470 (3)	C14—H14	0.9300
C2—H2C	0.9700	C15—N6	1.331 (3)
C2—H2D	0.9700	C15—H15	0.9300
C3—N3	1.460 (3)	C16—O3	1.230 (3)
C3—H3C	0.9700	C16—O4	1.240 (3)
C3—H3D	0.9700	N1—H1A	0.9000
C4—N4	1.485 (3)	N1—H1B	0.9000
C4—H4C	0.9700	N2—H2A	0.9000
C4—H4D	0.9700	N2—H2B	0.9000
C5—C9	1.393 (3)	N3—H3B	0.89 (3)
C5—C6	1.403 (3)	N3—H3A	0.87 (4)
C5—C10	1.497 (3)	N4—H4A	0.9000
C6—N5	1.324 (3)	N4—H4B	0.9000
C6—S1	1.7922 (19)	O1W—H1W	0.803 (17)
C7—N5	1.343 (3)	O1W—H2W	0.847 (17)
C7—C8	1.371 (3)	O2W—H3W	0.820 (18)
C7—H7	0.9300	O2W—H4W	0.830 (17)
C8—C9	1.368 (3)	O3W—H5W	0.828 (18)
C8—H8	0.9300	O3W—H6W	0.841 (18)
C9—H9	0.9300	S1—S2	2.0352 (8)
			
N1—Cu1—N1^i^	180.00 (12)	O1—C10—C5	118.17 (19)
N1—Cu1—N2^i^	96.00 (8)	O2—C10—C5	117.53 (17)
N1^i^—Cu1—N2^i^	84.00 (8)	N6—C11—C12	122.8 (2)
N1—Cu1—N2	84.00 (8)	N6—C11—S2	117.04 (16)
N1^i^—Cu1—N2	96.00 (8)	C12—C11—S2	120.17 (15)
N2^i^—Cu1—N2	180.0	C13—C12—C11	116.90 (19)
N3^ii^—Cu2—N3	180.00 (16)	C13—C12—C16	119.6 (2)
N3^ii^—Cu2—N4^ii^	95.42 (8)	C11—C12—C16	123.50 (19)
N3—Cu2—N4^ii^	84.58 (8)	C14—C13—C12	120.7 (2)
N3^ii^—Cu2—N4	84.58 (8)	C14—C13—H13	119.7
N3—Cu2—N4	95.42 (8)	C12—C13—H13	119.7
N4^ii^—Cu2—N4	180.00 (14)	C15—C14—C13	117.4 (2)
N1—C1—C2	106.56 (18)	C15—C14—H14	121.3
N1—C1—H1C	110.4	C13—C14—H14	121.3
C2—C1—H1C	110.4	N6—C15—C14	124.3 (2)
N1—C1—H1D	110.4	N6—C15—H15	117.8
C2—C1—H1D	110.4	C14—C15—H15	117.8
H1C—C1—H1D	108.6	O3—C16—O4	124.2 (2)
N2—C2—C1	107.41 (19)	O3—C16—C12	118.8 (2)
N2—C2—H2C	110.2	O4—C16—C12	117.0 (2)
C1—C2—H2C	110.2	C1—N1—Cu1	108.14 (14)
N2—C2—H2D	110.2	C1—N1—H1A	110.1
C1—C2—H2D	110.2	Cu1—N1—H1A	110.1
H2C—C2—H2D	108.5	C1—N1—H1B	110.1
N3—C3—C4^ii^	109.0 (2)	Cu1—N1—H1B	110.1
N3—C3—H3C	109.9	H1A—N1—H1B	108.4
C4^ii^—C3—H3C	109.9	C2—N2—Cu1	108.99 (13)
N3—C3—H3D	109.9	C2—N2—H2A	109.9
C4^ii^—C3—H3D	109.9	Cu1—N2—H2A	109.9
H3C—C3—H3D	108.3	C2—N2—H2B	109.9
N4—C4—C3^ii^	108.4 (2)	Cu1—N2—H2B	109.9
N4—C4—H4C	110.0	H2A—N2—H2B	108.3
C3^ii^—C4—H4C	110.0	C3—N3—Cu2	108.78 (15)
N4—C4—H4D	110.0	C3—N3—H3B	109.8 (18)
C3^ii^—C4—H4D	110.0	Cu2—N3—H3B	113.5 (18)
H4C—C4—H4D	108.4	C3—N3—H3A	108 (2)
C9—C5—C6	116.28 (19)	Cu2—N3—H3A	110 (2)
C9—C5—C10	119.46 (18)	H3B—N3—H3A	106 (3)
C6—C5—C10	124.26 (17)	C4—N4—Cu2	107.67 (15)
N5—C6—C5	123.55 (18)	C4—N4—H4A	110.2
N5—C6—S1	116.14 (15)	Cu2—N4—H4A	110.2
C5—C6—S1	120.31 (15)	C4—N4—H4B	110.2
N5—C7—C8	123.6 (2)	Cu2—N4—H4B	110.2
N5—C7—H7	118.2	H4A—N4—H4B	108.5
C8—C7—H7	118.2	C6—N5—C7	117.75 (19)
C9—C8—C7	117.90 (19)	C11—N6—C15	117.9 (2)
C9—C8—H8	121.0	H1W—O1W—H2W	109 (3)
C7—C8—H8	121.0	H3W—O2W—H4W	106 (3)
C8—C9—C5	120.92 (19)	H5W—O3W—H6W	111 (3)
C8—C9—H9	119.5	C6—S1—S2	102.41 (7)
C5—C9—H9	119.5	C11—S2—S1	103.30 (7)
O1—C10—O2	124.3 (2)		
			
N1—C1—C2—N2	−54.5 (2)	C11—C12—C16—O4	174.2 (3)
C9—C5—C6—N5	0.8 (3)	C2—C1—N1—Cu1	44.0 (2)
C10—C5—C6—N5	−179.42 (19)	N2^i^—Cu1—N1—C1	161.56 (15)
C9—C5—C6—S1	−179.23 (15)	N2—Cu1—N1—C1	−18.44 (15)
C10—C5—C6—S1	0.5 (3)	C1—C2—N2—Cu1	38.5 (2)
N5—C7—C8—C9	−0.7 (4)	N1—Cu1—N2—C2	−11.41 (15)
C7—C8—C9—C5	0.8 (3)	N1^i^—Cu1—N2—C2	168.59 (15)
C6—C5—C9—C8	−0.9 (3)	C4^ii^—C3—N3—Cu2	−38.3 (3)
C10—C5—C9—C8	179.36 (19)	N4^ii^—Cu2—N3—C3	12.94 (18)
C9—C5—C10—O1	1.9 (3)	N4—Cu2—N3—C3	−167.06 (18)
C6—C5—C10—O1	−177.85 (19)	C3^ii^—C4—N4—Cu2	39.1 (3)
C9—C5—C10—O2	−176.54 (19)	N3^ii^—Cu2—N4—C4	−14.58 (18)
C6—C5—C10—O2	3.7 (3)	N3—Cu2—N4—C4	165.42 (18)
N6—C11—C12—C13	1.5 (3)	C5—C6—N5—C7	−0.7 (3)
S2—C11—C12—C13	−178.95 (17)	S1—C6—N5—C7	179.35 (17)
N6—C11—C12—C16	−178.9 (2)	C8—C7—N5—C6	0.6 (3)
S2—C11—C12—C16	0.7 (3)	C12—C11—N6—C15	−1.5 (3)
C11—C12—C13—C14	−0.4 (4)	S2—C11—N6—C15	178.94 (19)
C16—C12—C13—C14	−180.0 (2)	C14—C15—N6—C11	0.4 (4)
C12—C13—C14—C15	−0.7 (4)	N5—C6—S1—S2	−9.32 (17)
C13—C14—C15—N6	0.7 (4)	C5—C6—S1—S2	170.74 (15)
C13—C12—C16—O3	174.7 (2)	N6—C11—S2—S1	−2.08 (18)
C11—C12—C16—O3	−4.9 (4)	C12—C11—S2—S1	178.35 (15)
C13—C12—C16—O4	−6.2 (4)	C6—S1—S2—C11	81.99 (10)

Symmetry codes: (i) −*x*, −*y*, −*z*+1; (ii) −*x*+1, −*y*, −*z*.

### Hydrogen-bond geometry (Å, °)

**Table:**

*D*—H···*A*	*D*—H	H···*A*	*D*···*A*	*D*—H···*A*
N1—H1A···O2^iii^	0.90	2.25	3.084 (3)	154
N1—H1B···O3	0.90	2.48	3.138 (3)	130
N2—H2A···O3W^iv^	0.90	2.38	3.213 (3)	154
N2—H2B···O4^i^	0.90	2.59	3.345 (4)	142
N4—H4B···O2^v^	0.90	2.27	3.116 (3)	157
O1W—H2W···O2^vi^	0.85 (2)	1.93 (2)	2.771 (2)	175 (3)
O1W—H1W···O3W	0.80 (2)	2.10 (2)	2.892 (3)	172 (3)
O2W—H3W···O4^i^	0.82 (2)	1.95 (2)	2.712 (3)	154 (4)
O2W—H4W···O1^vii^	0.83 (2)	2.08 (2)	2.897 (3)	168 (3)
O3W—H5W···O1^vii^	0.83 (2)	2.03 (2)	2.838 (3)	167 (3)
O3W—H6W···O3^viii^	0.84 (2)	1.98 (2)	2.812 (2)	170 (3)
N3—H3A···O4W	0.87 (4)	2.42 (3)	3.045 (4)	129 (3)

Symmetry codes: (iii) −*x*, −*y*+1, −*z*+1; (iv) −*x*+1, −*y*, −*z*+1; (i) −*x*, −*y*, −*z*+1; (v) *x*, *y*−1, *z*−1; (vi) *x*, *y*−1, *z*; (vii) −*x*+1, −*y*+1, −*z*+1; (viii) *x*+1, *y*, *z*.
